# Histone Demethylation Profiles in Nonalcoholic Fatty Liver Disease and Prognostic Values in Hepatocellular Carcinoma: A Bioinformatic Analysis

**DOI:** 10.3390/cimb45040237

**Published:** 2023-04-20

**Authors:** Yuanbin Liu, Mingkai Chen

**Affiliations:** Department of Gastroenterology, Renmin Hospital of Wuhan University, No. 99 Zhang Zhidong Road, Wuhan 430000, China; 2016302180347@whu.edu.cn

**Keywords:** nonalcoholic fatty liver disease, epigenetics, histone demethylation, hepatocellular carcinoma, bioinformatics

## Abstract

Nonalcoholic fatty liver disease (NAFLD) is the most common chronic liver disease with multifactorial pathogenesis; histone demethylases (HDMs) are emerging as attractive targets. We identified HDM genes (including KDM5C, KDM6B, KDM8, KDM4A, and JMJD7) that were differentially expressed in NAFLD and normal samples by exploring gene expression profiling datasets. There was no significant difference in the expression of genes related to histone demethylation between mild and advanced NAFLD. In vitro and in vivo studies indicated that KDM6B and JMJD7 were upregulated at the mRNA level in NAFLD. We explored the expression levels and prognostic values of the identified HDM genes in hepatocellular carcinoma (HCC). KDM5C and KDM4A were upregulated in HCC compared to normal tissue, while KDM8 showed downregulation. The abnormal expression levels of these HDMs could provide prognostic values. Furthermore, KDM5C and KDM4A were associated with immune cell infiltration in HCC. HDMs were associated with cellular and metabolic processes and may be involved in the regulation of gene expression. Differentially expressed HDM genes identified in NAFLD may provide value to understanding pathogenesis and in the development of epigenetic therapeutic targets. However, on the basis of the inconsistent results of in vitro studies, future in vivo experiments combined with transcriptomic analysis are needed for further validation.

## 1. Introduction

Nonalcoholic fatty liver disease (NAFLD) is a pathological condition in which fat accumulates in the liver without excessive alcohol consumption and other causes of liver disease [1]. NAFLD is a dynamic and progressive process, with the disease spectrum ranging from simple steatosis (SS) to the more severe nonalcoholic steatohepatitis (NASH), characterized by hepatocyte ballooning and liver inflammation, and a subset of patients could progress to cirrhosis and hepatocellular carcinoma (HCC) [2,3]. NAFLD is the most common chronic liver disease, with a global adult prevalence of approximately 25%, and is emerging as a major cause of cirrhosis and HCC [1,4,5]. Given that NAFLD is a serious public health concern, and the disease burden is still expected to increase [6], a deeper understanding of the pathogenic dimension would support controlling this epidemic.

Epigenetics is defined as heritable alterations in gene expression [7]. Multiple factors such as the environment, diet, and lifestyle could modulate epigenetics and, thus, impact gene expression [7,8]. The role that epigenetic modifications play and the patterns that they follow in the pathogenesis of NAFLD have been investigated. Epigenetic regulation in NAFLD mainly comprises DNA methylation, histone modifications, and noncoding RNAs [9,10,11]. Histone modifications are mediated via the transfer or removal of amino acid residues from the histone tails by some enzymes [12]. As a form of histone modification, histone demethylation activates or represses gene expression by removing methyl groups from modified histones through histone demethylases (HDMs). HDMs are classified as lysine-specific demethylases (LSDs) and Jumonji domain-containing (JMJD) demethylases [13]. These histone lysine demethylases (KDMs) have been partially studied in animal models and cell lines in NAFLD. KDM7A was overexpressed in mice and the alpha mouse liver 12 (AML12) cell lines and induced hepatic steatosis [14]. Plant homeodomain finger 2 (Phf2 or KDM7C) protected the liver from oxidative stress and fibrogenesis in a mouse model of NAFLD through the demethylation of histone H3 lysine 9 demethylation (H3K9me2) at the promoter of carbohydrate responsive element binding protein (ChREBP) [15]. Another histone, H3K9 demethylase JMJD2B, with increased expression in in vivo and in vitro models of NAFLD promoted hepatic steatosis by upregulating ligand-activated liver X receptor α (LXRα)-independent lipogenic genes [16] and peroxisome proliferator-activated receptor γ2 (PPARγ2) [17].

The role of histone demethylation in regulating lipid-metabolism gene expression in NAFLD has led to emerging promising insights into pathogenesis and therapeutic targets. There is no specific description of histone demethylation profiles in NAFLD. Here, we use databases to explore the expression changes in histone–demethylation-related genes in NAFLD and their role in HCC to obtain promising epigenetic targets. We also probed interaction genes and relevant protein–protein interaction (PPI) networks and predicted the biological processes and pathways involved in these proteins.

## 2. Methods

### 2.1. Dataset Acquisition

The Gene Expression Omnibus (GEO) database (https://www.ncbi.nlm.nih.gov/gds/, accessed on 14 May 2022) was queried with search terms ‘nonalcoholic fatty liver’ and ‘homo sapiens’ to identify genes encoding histone demethylation in NAFLD. The inclusion criteria for the dataset were a sample size of at least 20, the diagnosis of NAFLD being a liver biopsy, and raw data being available from GEO. Two datasets, GSE89632 and GSE49541, were lastly included. The GSE89632 dataset consisted of 63 participants: 19 SS patients, 20 NASH patients, and 24 healthy controls (HCs) [18]. The platform for this dataset was GPL14951, and the assay technology used was the Illumina HumanHT-12 WG-DASL V4.0 R2 expression beadchip. The GSE49541 dataset included 72 patients with NAFLD, 40 of whom had mild NAFLD (Fibrosis Stage 0–1) and 32 with advanced NAFLD (Fibrosis Stage 3–4) [19,20,21]. The platform for this dataset was GPL570, and the used chip was the Affymetrix Human Genome U133 Plus 2.0 Array.

### 2.2. Differentially Expressed Gene Identification

Differentially expressed genes (DEGs) were determined using the ‘limma’ package in R software [22]. The filtering criteria were |log2 fold change (FC)| > 0.5 and an adjusted *p*-value < 0.05. The heatmaps for the DEGs were created using the ‘pheatmap’ package (https://www.rdocumentation.org/packages/pheatmap/, accessed on 26 May 2022) in R software. The volcano plots were outlined using the ‘ggrepel’ package (https://www.rdocumentation.org/packages/ggrepel/, accessed on 26 May 2022) in R. In the GSE89632 dataset, DEGs were obtained via SS vs. HC, NASH vs. HC, and NASH vs. SS. In the GSE49541 dataset, DEGs were accessed by comparing mild and advanced NAFLD.

### 2.3. Retrieving the Genes Encoding HDM

Genes encoding HDM were manually retrieved from the obtained DEGs, and those fulfilling an adjusted *p*-value < 0.05 but not reaching |log2 FC| > 0.5 were also recorded. Differential expression box plots for DEGs in HDM were mapped using the ‘ggplot2’ package (https://rdocumentation.org/packages/ggplot2/, accessed on 26 May 2022) in R.

### 2.4. In Vitro Model Validation

We established NAFLD models using human normal liver cell line L-02 (Pricella, Wuhan, China, HL-7702) and oleic acid (GLPBIO, USA, GC30110) to validate the identified DEGs. Oleic acid is a widely used reagent for constructing experimental NAFLD in in vitro cell models, and we explored the optimal concentration for inducing NAFLD. After confirming successful modeling by oil red O staining (Servicebio, Wuhan, China, G1015), we extracted RNA from experimental and control groups and detected gene expression by quantitative real-time polymerase chain reaction (qRT-PCR). First, 1 mL of Trizol (Thermo, Waltham, MA, USA, 15596026) was added to the cells and lysed for 10 min at room temperature. Next, 200 µL of chloroform (Sinopharm Chemical Reagent Company, Shanghai, China, 10006818) was added, shaken, and mixed, and then left to stand at room temperature for 5 min, followed by a 4 °C centrifuge at 12,000 r/min for 10 min to form a stratified layer with DNA and protein in the lower layer and RNA-containing water in the upper layer. We then aspirated the water phase into a new 1.5 mL tube, added an equal volume of isopropanol (Sinopharm Chemical Reagent Company, Shanghai, China, 80109218), mixed it upside down, and left it to stand at room temperature for 10 min, then centrifuged at 4 °C for 10 min at 12,000 r/min. The supernatant was then discarded, and the white precipitate was the RNA. Finally, the desired RNA is dissolved by removing impurities and adding 20 µL of RNase-free water to the precipitate. This was followed by a reverse transcription kit (Servicebio, Wuhan, China, G3330-100) to synthesize cDNA. Our qRT-PCR protocol was as the following: take 0.2 mL PCR tubes and prepare the following reaction system including 2 × SYBR Green qPCR Master Mix (Servicebio, Wuhan, China, G3321-15) 10 μL, Forward Primer and Reverse Primer (Sangon Biotech, Shanghai, China) 0.4 μL respectively, cDNA template 1.0 μL and nuclease-free water (Solarbio, Beijing, China, 3209 was added to 20ul. The PCR amplification was performed at a PCR amplifier (TELSTAR BIO-II-A, Mx300P): pre-denaturation 95 °C for 10 min; cycling (40 times) 95 °C for 15 s, 62 °C for 30 s, 72 °C for 30 s; melting curve from 60 °C to 95 °C, with a 0.5 °C temperature increase every 15 s. The forward and reverse primers for the corresponding genes, including KDM5C, KDM6B, KDM8, KDM4A, and JMJD7, were summarized in Table 1.

### 2.5. HDM Gene Expression Levels and Prognostic Value in HCC

We explored the expressions of identified genes in HCC and investigated their expressions as altered compared with normal tissues. We also identified genes with prognostic values for HCC. The Gene Expression Profiling Interactive Analysis (GEPIA) database is a new web server using RNA sequencing expression data from The Cancer Genome Atlas (TCGA) and Genotype-Tissue Expression (GTEx) for 9736 tumor tissues and 8587 normal tissue samples. We accessed the GEPIA database (http://gepia.cancer-pku.cn/, accessed on 28 May 2022) to characterize the expression profiles and to evaluate the prognostic values of selected HDM genes in HCC [23]. The Human Protein Atlas (HPA) database (https://www.proteinatlas.org/, accessed on 29 May 2022) was used to explore the immunohistochemical (IHC) information on HDM gene expression [24,25,26]. We also investigated the association of HDM gene expression with immune cell infiltration in HCC using the TIMER database (http://cistrome.org/TIMER/, accessed on 8 June 2022) [27].

### 2.6. Interactive Gene and PPI Network Construction

We used the genemania database (http://genemania.org/, accessed on 8 June 2022) to map genes that interact with differentially expressed HDM genes and also explore functional interactions [28]. We then determined the proteins that could interact with HDMs to shed light on the functional links of other proteins with HDMs. We constructed PPI networks of identified HDM genes using the STRING database (https://cn.string-db.org/, accessed on 8 June 2022) [29]. Finally, we identified 30 proteins that interact with these HDMs and then obtained Gene Ontology (GO) analysis and pathway and process enrichment analysis of the genes corresponding to these interacting proteins through the Metascape database (https://metascape.org/, accessed on 8 June 2022) [30].

### 2.7. Statistical Analysis

All bioinformatic analysis was performed using R software version 4.1.3. The Kruskal–Wallis test was carried out to compare the differential expression of HDM genes among SS, NASH, and HC. The Wilcoxon test was utilized to compare differences in expression between the two groups in the data set GSE49541. We used GraphPad Prism 8.3 software for statistical analysis of mRNA expressions in vitro. In vitro mRNA expression levels were expressed as mean and standard deviation, and an unpaired t-test was used for statistical analysis. The *p*-value < 0.05 was considered statistically significant. *p* < 0.05 was signified as *, *p* < 0.01 as **, *p* < 0.001 as ***, and NS indicated not statistically significant.

## 3. Results

### 3.1. DEGs Identification in NAFLD

**Figure 1:**
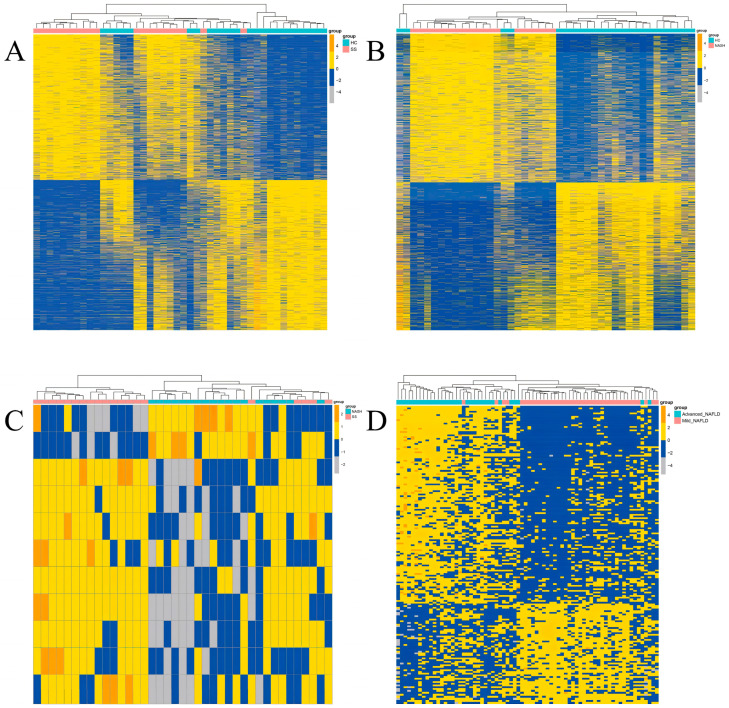
Heatmaps of the DEGs for both databases. (**A**) Comparison of SS and HC in the GSE89632 database. (**B**) Comparison of NASH and HC in the GSE89632 database. (**C**) Comparison of NASH and SS in the GSE89632 database. (**D**) Advanced NAFLD compared to mild NAFLD in the GSE49541 database. Abbreviations: DEGs, differentially expressed genes; SS, simple steatosis; HC, healthy control; NASH, nonalcoholic steatohepatitis; NAFLD, nonalcoholic fatty liver disease.

There were 29,377 genes included in the GSE89632 dataset, leaving 20,818 unique genes after removing duplicates. Upregulated genes were described as |log2 FC| > 0.5 combined with adjusted *p*-values < 0.05, while downregulated genes were specified as |log2 FC| < −0.5 and adjusted *p*-values < 0.05. For the comparison between SS and HC, 1532 and 1580 genes were up- and down-regulated, respectively. A total of 2699 DEGs were identified in contrast between NASH and HC samples, of which 1355 and 1344 genes were revealed to be up- and downregulated, respectively. However, only 11 DEGs were recorded regarding the NASH vs. SS samples, with 2 upregulated genes and 9 downregulated genes. Using the GSE49541 dataset, we contrasted the respective gene expression alterations in advanced NAFLD and mild NAFLD and found that only 175 DEGs in total were identified under the cut-off criteria, of which 117 genes were upregulated and 58 genes were downregulated in advanced NAFLD. The heatmaps and volcano plots for both datasets are shown in Figure 1 and Figure S1.

### 3.2. Differentially Expressed HDM Genes

**Figure 2:**
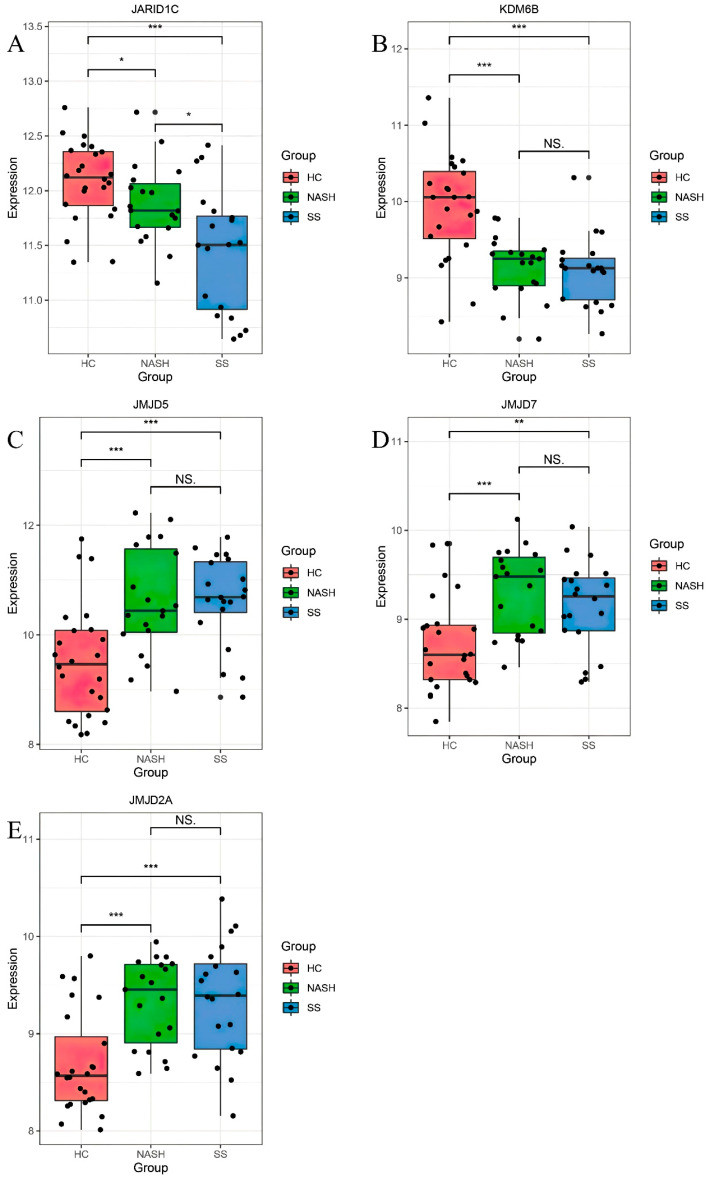
Differentially expressed HDM genes in the GSE89632 dataset. Abbreviations: SS, simple steatosis; HC, healthy control; NASH, nonalcoholic steatohepatitis; HDM, histone demethylase. *p* < 0.05 was signified as *, *p* < 0.01 as **, *p* < 0.001 as ***, and NS indicated not statistically significant.

We ascertained the HDM genes that were differentially expressed in the grouped sample comparisons, and those that met only an adjusted *p*-value of < 0.05 were also recorded. In the SS and HC comparisons, KDM2B, KDM6B, JARID1C (also referred to as KDM5C), ALKBH1, JMJD5 (also referred to as KDM8), JMJD6, JMJD7, JMJD2A (also referred to as KDM4A) fulfilled an adjusted *p*-value < 0.05, and JARID1C, KDM6B, JMJD5, and JMJD2A were identified as DEGs. In NASH and HC samples, the differential expression of KDM6B, KDM4D, JMJD5, JMJD6, JMJD7, and JMJD2A demonstrated statistical significance, and among them, KDM6B, JMJD5, JMJD7, and JMJD2A acting as DEGs. Interestingly, no HDM encoding genes were expressed significantly differently in NASH vs. SS samples (all adjusted *p*-values > 0.05) (Figure 2). Similarly, in the GSE49541 dataset, there were no differentially expressed HDM genes were derived in samples with advanced NAFLD and mild NAFLD (all adjusted *p*-values > 0.05) (Figure S2).

Collectively, a total of five HDM genes were identified as DEGs in NAFLD, with JMJD5, JMJD7, and JMJD2A exhibiting increased expression compared with HC, while JARID1C and KDM6B were downregulated. KDM6B, JMJD5, and JMJD2A were DEGs in both SS vs. HC and NASH vs. HC. JMJD5 manifested the highest |log2 FC| values for differential expression, which exceeded 1 in both SS and NASH samples compared with HC.

### 3.3. In Vitro Validation

**Figure 3:**
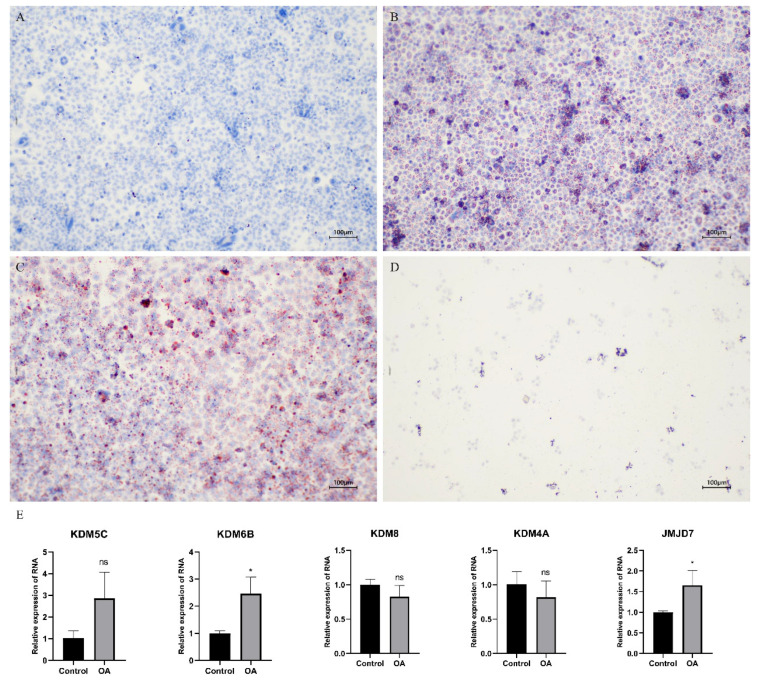
In vitro studies to verify gene expression. (**A**) Not treated with oleic acid. (**B**) L-02 cells treated with 0.5 mM oleic acid (Oil Red O staining). (**C**) L-02 cells treated with 1 mM oleic acid. (**D**) L-02 cells treated with 2 mM oleic acid. (**E**) qRT-PCR to verify gene expression. Abbreviations: qRT-PCR, quantitative real-time polymerase chain reaction; OA, oleic acid; ns, not significant. An asterisk (*) indicates that the *p*-value of < 0.05.

After comparing multiple oleic acid concentrations, we noted that cells at 0.5 mM exhibited optimal lipid accumulation, while cells at 1.0 mM and 2.0 mM presented cell death. Therefore, we chose 0.5 mM oleic acid to construct the NAFLD model and demonstrated the lipid accumulation by oil red O staining. The qRT-PCR results showed that only KDM6B and JMJD7 demonstrated statistically significant upregulation, while other gene expressions did not differ between groups (Figure 3).

### 3.4. HDM Gene Expression and Prognostic Values in HCC

**Figure 4:**
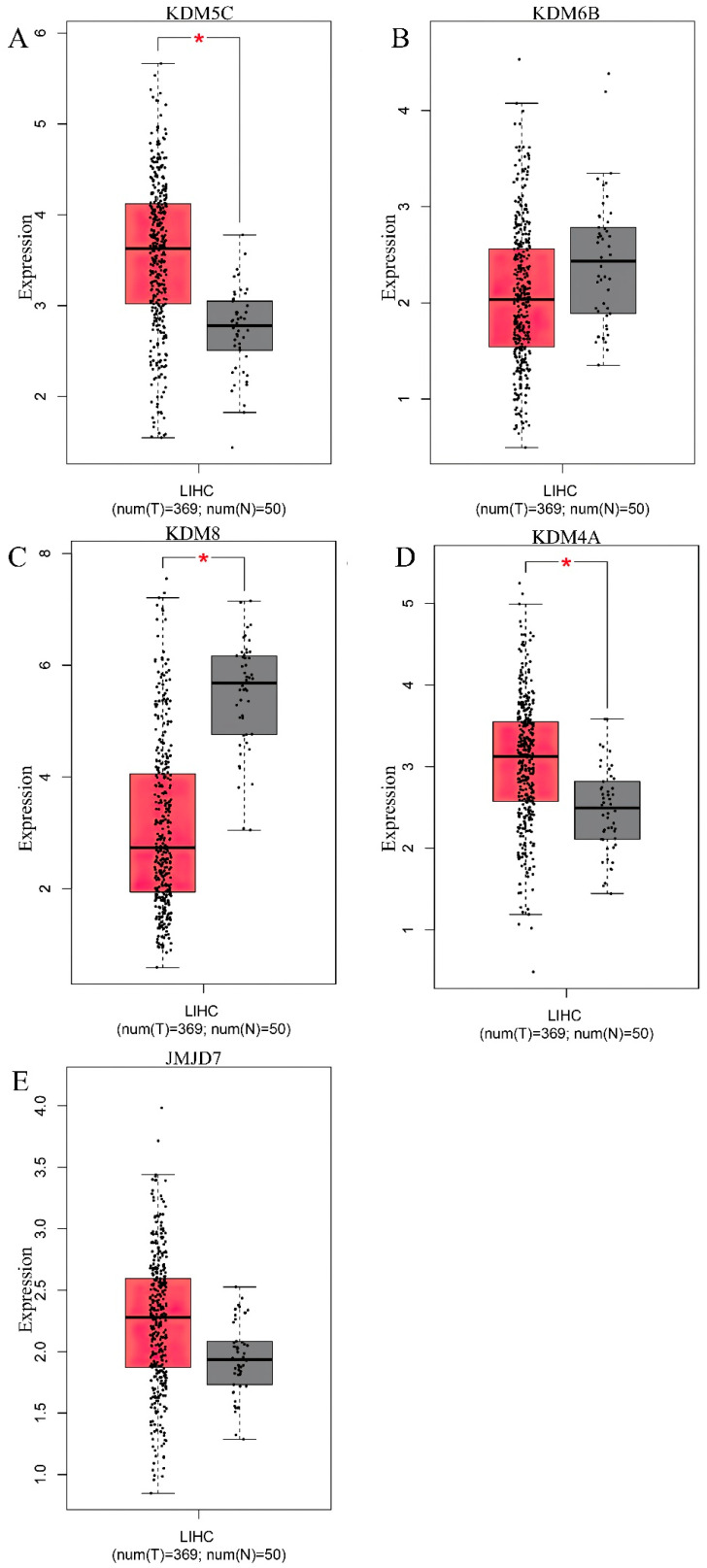
HDM gene expression in HCC versus normal tissues. (**A**) KDM5C. (**B**) KDM6B. (**C**) KDM8. (**D**) KDM4A. (**E**) JMJD7. * Implies statistically significant difference. Abbreviations: HCC, hepatocellular carcinoma; LIHC, liver hepatocellular carcinoma. An asterisk indicates that the *p*-value of <0.05.

**Figure 5:**
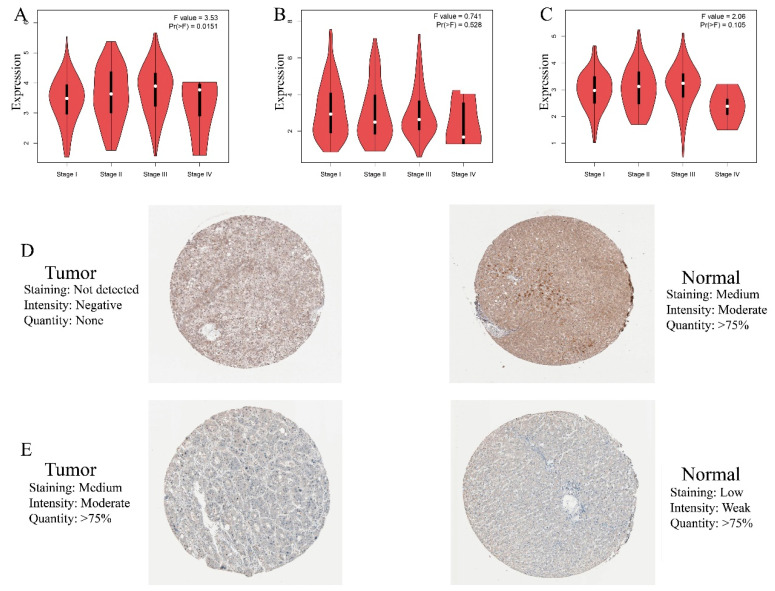
Association of KDM5C, KDM8, and KDM4A expression with HCC staging and accessible immunohistochemical data for protein expression. (**A**) Association of KDM5C expression with HCC staging. (**B**) Association of KDM8 expression with HCC staging. (**C**) Association of KDM4A expression with HCC staging. (**D**) IHC of KDM8 expression in HPA. (**E**) IHC of KDM4A expression in HPA. Abbreviations: HCC, hepatocellular carcinoma; HPA, The Human Protein Atlas.

We first queried the expression levels of these five HDM genes in HCC using the GEPIA database. We included matching TCGA normal tissues as controls and selected the corresponding tumor type of liver hepatocellular carcinoma (LIHC) in TCGA. The screening criteria were also |log2 FC| > 0.5 and *p*-value cut-off < 0.05. KDM5C (Figure 4A), KDM8 (Figure 4C), and KDM4A (Figure 4D) were differentially expressed in HCC compared to normal tissue, with KDM8 showing the most remarkable difference (|log2 FC| > 1). No significant difference in the expression of KDM6B (Figure 4B) and JMJD7 (Figure 4E) was observed. KDM5C and KDM4A expressions were increased in HCC, while KDM8 was reduced. KDM5C (Figure 5A), but not KDM8 (Figure 5B) and KDM4A (Figure 5C), was associated with HCC staging. We also validated the protein expressions of KDM8 and KDM4A in HCC at the protein level using IHC data from the HPA database. KDM8 staining in HCC tissues was from undetectable to medium, and the intensity ranged from negative to moderate, as compared to medium and moderate in normal tissues, respectively. (Figure 5D) The protein expression level of KDM4A in HCC was upregulated compared to normal tissues based on IHC (Figure 5E).

**Figure 6:**
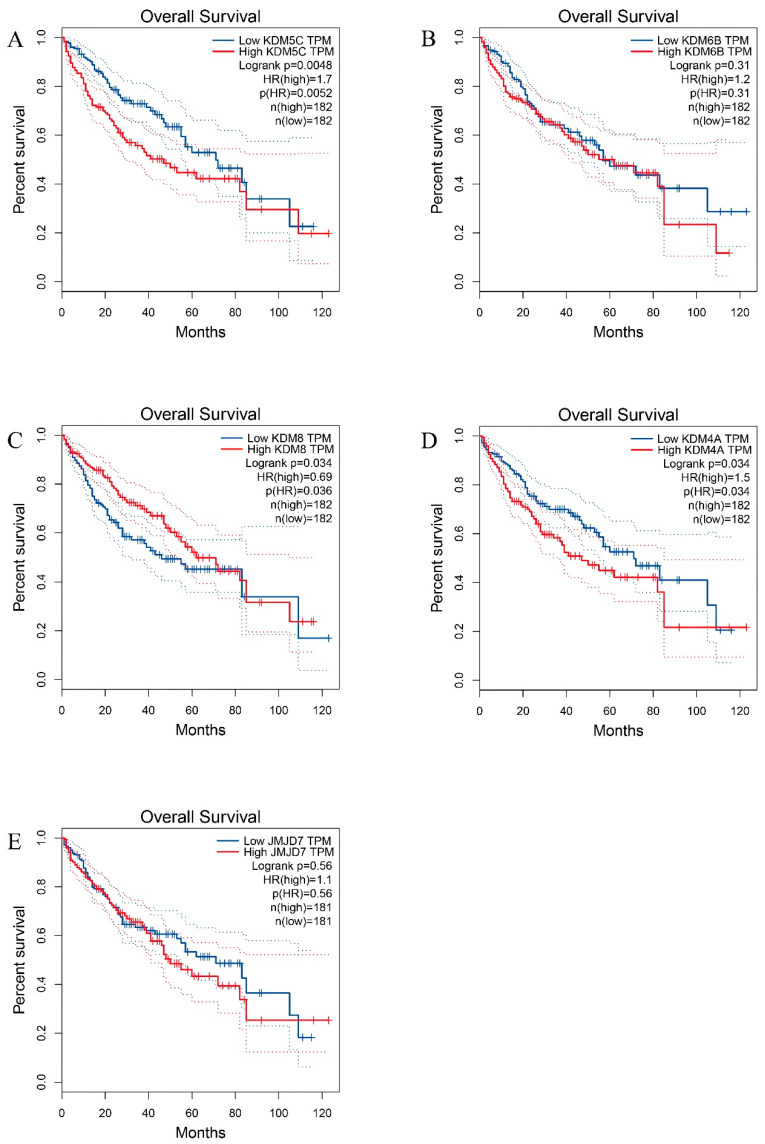
Prognostic values of five HDM genes for overall survival in HCC. (**A**) KDM5C. (**B**) KDM6B. (**C**) KDM8. (**D**) KDM4A. (**E**) JMJD7. Abbreviations: HCC, hepatocellular carcinoma; TPM, Transcripts Per Million; HR, hazard ratio.

**Figure 7:**
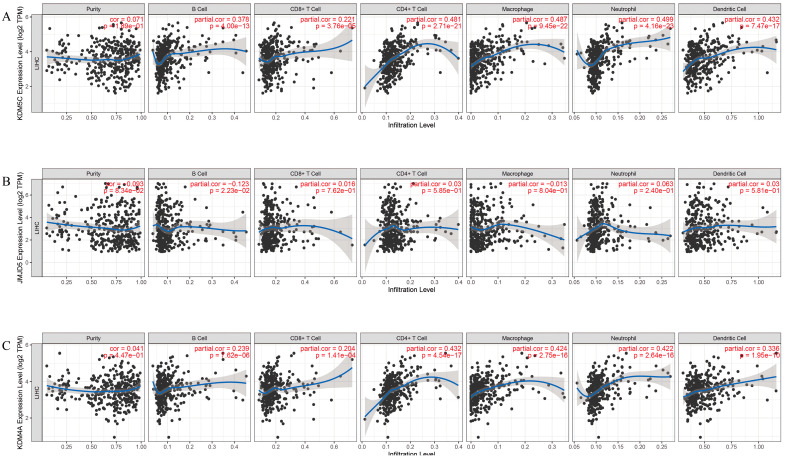
Association of KDM5C, KDM8, and KDM4A with immune cell infiltration in the TIMER database. (**A**) KDM5C. (**B**) KDM8. (**C**) KDM4A. Abbreviations: TPM, Transcripts Per Million; LIHC, liver hepatocellular carcinoma.

We then addressed the prognostic values of these genes in HCC. Using the GEPIA database, we revealed that KDM5C (Figure 6A), KDM8 (Figure 6C), and KDM4A (Figure 6D) were predictive genes for overall survival (OS), while only KDM5C (Figure S3) had prognostic value for disease-free survival (RFS) in HCC. Furthermore, KDM5C and KDM4A were correlated with immune infiltration in HCC, whereas KDM8 showed no noticeable differences (Figure 7).

### 3.5. Interaction Genes and PPI Network

**Figure 8:**
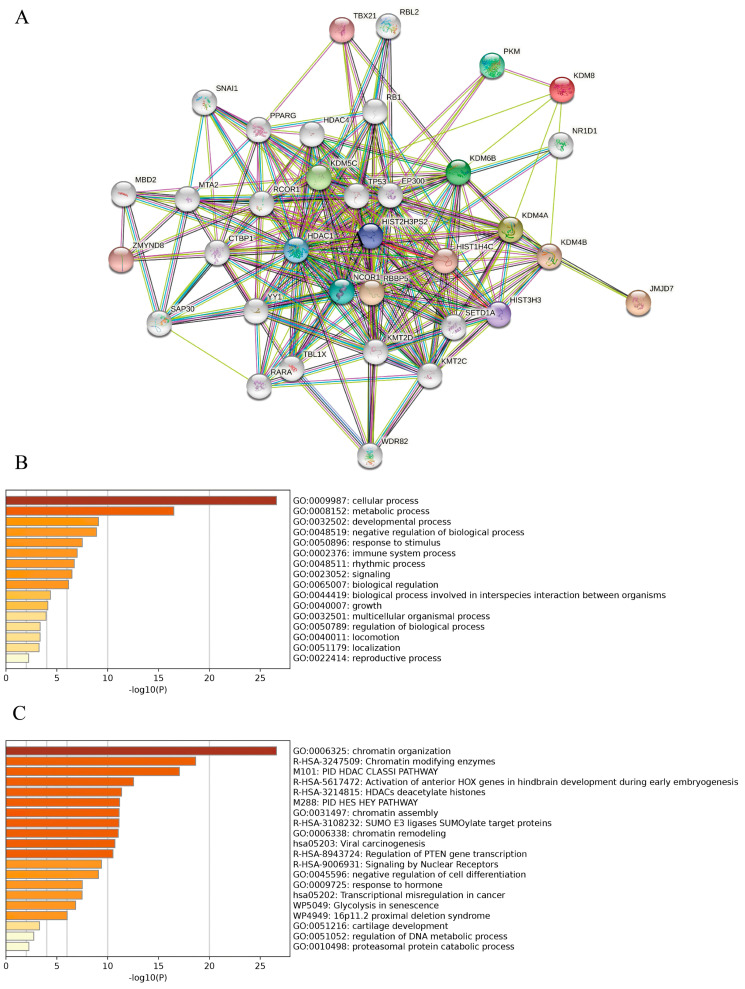
The PPI network of identified HDM genes and Gene Ontology (GO) analysis and pathway and process enrichment analysis of interacting proteins using Metascape. (**A**) PPI network of five HDMs. (**B**) GO biological processes analysis of interacting proteins. (**C**) Pathway and process enrichment analysis of the top 20 clusters. Abbreviations: PPI, protein–protein interaction; HDM, histone demethylase.

We next utilized the genemania database to obtain genes that could interact with these genes and to reveal the functional connectivity among them. A total of 20 most likely interacting genes and 239 linkages were established among these HDM genes. Notably, we revealed that JMJD7 exhibited no function, including demethylase activity. The five most relevant genes were *FOS*, *JUN*, *DRG2*, *EPHB2,* and *BRD4*. *FOS*, *DRG2*, *EPHB2*, and *BRD4* with KDM5C, *JUN* with KDM4A, and *EPHB2* with KDM6B were co-expressed. *FOS*, *JUN*, *BRD4* with KDM6B, *DRG2*, *EPHB2* with JMJD7, and *EPHB2* with KDM4A were physically linked. *BRD4* was genetically linked to KDM5C and KDM6B. The strongest functional links were HDM activity and protein demethylase activity, both found among other HDMs. (Figure S4) Using the STRING database, we explored the interactions of these five HDMs with other proteins. We identified 30 interacting proteins in the PPI network. (Figure 8A) Top-level GO analysis of biological processes indicated that cellular processes and metabolic processes were the most prominent processes. (Figure 8B) The pathway and process enrichment analysis revealed the most common clusters to be chromatin organization and chromatin modifying enzymes (Figure 8C).

## 4. Discussion

The development and progression of NAFLD are multifactorial and multidimensional processes in which genetic and epigenetic factors represent instrumental drivers. Epigenetic regulation can govern the expression of metabolic-related genes in NAFLD by switching key genes on or off. Therefore, insights into the involved profiles would be of significant relevance to the understanding of NAFLD and the development of potential targets. Histone demethylation as a type of histone post-transcriptional modification could modify the gene expression patterns through the removal of methylation marks on histones. In this study, we compared whole gene expression differences between NAFLD and normal samples and between mild and advanced NAFLD by eliciting two GEO datasets, respectively. In the analysis of DEGs in SS vs. HC samples, we identified four genes related to histone demethylation, namely JARID1C (KDM5C), KDM6B, JMJD5 (KDM8), and JMJD2A (KDM4A). In the comparison of NASH with HC, we established that KDM6B, JMJD5, JMJD7, and JMJD2A are differentially expressed. In contrast, there was no significant difference in the expression of genes encoding HDM during disease progression in NAFLD in the two datasets. These findings may suggest that histone demethylation in NAFLD is not strongly associated with the degree of fibrosis or disease severity at least in the current bioinformatic explorations. However, based on the lack of compelling power of this exploration, future transcriptomic analyses are needed to uncover underlying the landscapes.

Although we identified five DEGs based on the screening criteria, there is currently very little description of their expressions in NAFLD or NASH. KDM5C is a histone H3K4-specific demethylase that has been shown to regulate fatty acid metabolism in several conditions. A recent study found that alcohol specifically activated KDM5B and KDM5C in male mice, resulting in decreased expression of hepatocyte nuclear factor 4 alpha, and could promote hepatocyte dedifferentiation and fatty acid synthesis. Knockdown of KDM5B and KDM5C prevented alcohol-induced lipid accumulation in male mice [31]. KDM5C was significantly downregulated in intrahepatic cholangiocarcinoma and served as a tumor suppressor by inhibiting cell proliferation, invasion, and fatty acid metabolism. Mechanistically, KDM5C exerted effects on tumor development by reducing H3K4me3 at specific promoters and downregulating its target gene fatty acid synthase (*FASN*) [32].

Currently, only expression alterations of KDM6B in fasting animal models and human samples of NAFLD have been described. KDM6B (also known as JMJD3) has been implicated as an epigenetic regulator in metabolic disorders, including NAFLD, and could specifically serve as a demethylase of histone H3K27. KDM6B is involved in several metabolic processes, such as lipolysis, glucose homeostasis, and insulin sensitivity [33,34]. Liver-specific downregulation of KDM6B resulted in intrinsic mitochondrial β-oxidation defects, which further led to hepatic steatosis and insulin and glucose intolerance. In fasting-induced mice, KDM6B functioned as a transcriptional regulator synergistically with sirtuin 1 (*SIRT1*) to activate β-oxidation but not gluconeogenic genes [35]. Therefore, KDM6B may be promising as an epigenetic target for the treatment of metabolic disorders, which could specifically address lipid levels without affecting glucose levels. In a recent study, both KDM6B and islet1 (*ISL1*) expressions were reduced in NAFLD, and they synergistically inhibited adipogenesis while promoting lipolysis. These effects were achieved because KDM6B could act on the methylation marks of the promoter on snail family transcriptional repressor 1 (*SNAI1*) [36]. However, this study similarly used bioinformatics analysis to determine that KDM6B expression is downregulated in NAFLD, but without demonstration by experimental models. In both in vivo and in vitro models, ISL1/KDM6B/SNAI1 axis was revealed to improve NAFLD by regulating lipid metabolism. Moreover, KDM6B was also involved in defective autophagy and hepatic steatosis in NAFLD. KDM6B may induce lipid degradation by epigenetically upregulating autophagic network genes. The mRNA and protein expressions of KDM6B were significantly reduced in human NAFLD samples compared to the normal population, and fibroblast growth factor-21 (*FGF21*) improved hepatic autophagy and lipolysis through KDM6B in NAFLD mice [37].

KDM8 is enriched in the liver tissue as a demethylase of histone H3K36me2 and is primarily involved in metabolism. We revealed in this study that the expression of KDM8 was significantly higher in NAFLD than in normal tissues and had the highest |log2 FC|. KDM8 was demonstrated to interact with pyruvate kinase muscle isozyme (PKM2) in cancer cells, impairing the tetramerization of PKM2 and blocking pyruvate kinase activity [38]. Furthermore, the interaction could regulate the nuclear translocation of PKM2 and promote the transactivation of hypoxia-inducible factor-1α (HIF-1α). Wang et al. also revealed that KDM8 was upregulated by hypoxia and involved in hypoxia-induced cell proliferation [38]. Knockdown of KDM8 inhibited the transcription of target genes of PKM2-HIF-1α involved in glucose metabolism, which reduced the glucose uptake and lactate secretion in cancer cells. It was likely that hypoxia similarly induces the upregulation of KDM8 expression in NAFLD, although available concrete descriptions were lacking. A study uncovered that G protein suppressor 2 (GPS2) can stabilize KDM4A from degradation by inhibiting ring finger protein 8 (RNF8) in adipocytes [39]. GPS2/KDM4A activity was essential for PPARγ-mediated specific transcriptional regulation. Stabilization of KDM4A by GPS2 enabled chromatin remodeling of PPARγ target genes such as lipolytic enzymes adipose triglyceride lipase (*ATGL*), hormone-sensitive lipase (*HSL*), and restin (*RETN*). KDM4A performed demethylation of histone H3K9 at the promoters of these key lipid metabolism genes for transcriptional regulation and was proved as critical for PPARγ-dependent gene activation. Finally, JMJD7 has been scarcely studied, and recently, it was suggested that it may be closer to Jumonji-C (JmjC) hydroxylase than to JmjC demethylase [40]. This was consistent with our finding that JMJD7 demonstrated no demethylase activity.

We then verified the changes in mRNA expression of these five genes compared to untreated controls by constructing NAFLD cell models in vitro. However, only KDM6B and JMJD7 were found to be upregulated. The mRNA expression trend of KDM6B in an in vitro NAFLD model was contrary to the results of our bioinformatic exploration. Available data indicated that the protein level of KDM6B in fasted mice was increased in the nucleus but decreased at the cytoplasmic level [35]. The mRNA and protein levels of KDM6B were also demonstrated to be reduced in samples of patients with NAFLD [37]. We suggested several reasons that could explain the difference. First, the level of our validation was at in vitro cellular model, which was notably dissimilar to that in human samples. In vitro studies are unable to model molecular and cell type interactions, and therefore, this may have led to different results since KDM6B may interact with multiple factors. Second, the cell line we used was L-02, and the NAFLD model was constructed using 0.5 mM oleic acid; although our cell line and oleic acid concentration were effective in inducing NAFLD, different cell lines and oleic acid concentrations may result in varying gene expression levels. In addition, there is no relevant literature demonstrating the altered expression of KDM6B in NAFLD in vitro or in vivo. Although liver-specific downregulation of KDM6B could trigger hepatic steatosis, the evidence for changes in KDM6B expression in experimental NAFLD is still lacking. We suggested here that the mRNA level of KDM6B was upregulated using an in vitro model, implying that KDM6B expression may be varied depending on the underlying conditions. However, KDM6B has nevertheless been proven to be a regulator involved in the development of NAFLD; thus, our results are simply more indicative of model specificity in its expression. Consistently, in vitro results revealed that JMJD7 was upregulated in NAFLD, but since it is more closely related to hydroxylase and was poorly studied, we would not discuss it further.

We have also discussed the expressions and prognostic values of these genes in HCC. KDM5C, KDM8, and KDM4A were notably differentially expressed in HCC compared to normal tissues. KDM5C was significantly upregulated in HCC and was identified as a prognostic gene for OS and RFS in HCC patients in this study. KDM5C, as a cancer driver gene in HCC, could have crosstalk of histone demethylation and metabolic reprogramming [41]. In a recent study, KDM5C was significantly upregulated in HCC in vitro, and silencing of KDM5C reduced the malignant behaviors of the cell lines and inhibited tumor growth in nude mice. KDM5C promoted HCC development by epigenetically downregulating inter-alpha-trypsin inhibitor heavy chain 1 (*ITIH1*) and thereby activating the PI3K/AKT pathway [42]. Another study indicated that KDM5C could facilitate cell migration, invasion, and epithelial–mesenchymal transition in HCC by inhibiting bone morphogenetic protein 7 (*BMP7*) expression [43]. KDM8 had the highest differential expression in this study and could be a predictor of OS, although of no value for RFS and immune infiltration. KDM8 was downregulated in HCC and may promote cell proliferation and in vivo tumorigenicity in HCC by accelerating the G1/S phase transition of the cell cycle [44]. KDM8 silencing also downregulated cyclin-dependent kinase inhibitor 1A (*CDKN1A*) expression in cell lines [44]. Therefore, KDM8 could function as a tumor suppressor gene in HCC and may perform histone H3K36 demethylation activity by binding to the *CDKN1A* promoter. KDM4A was upregulated in HCC tissues and could be predictive of OS. KDM4A was identified as a downstream target gene of regulatory factor X-5 (*RFX5*). *RFX5*/KDM4A exhibited pro-cancer effects by promoting the G0/G1 to S phase transition of the cell cycle in HCC and was also revealed to act on *p53* and downstream genes to inhibit cancer cell apoptosis [45].

We next identified genes that interacted most strongly with the indicated HDM genes. *FOS*, *JUN*, and *EPHB2* as proto-oncogenes may be involved in the role of KDM5C and KDM4A in HCC. *DRG2* and *BRD4* have regulatory roles in the modulation of cell growth and differentiation and could contribute to the role of selected HDM genes in NAFLD and HCC. We also determined 30 interacting proteins, with cellular and metabolic processes being the most significant in the GO biological process analysis, which may suggest that proteins associated with HDM are similarly involved in metabolic processes, further demonstrating the key role of HDMs as metabolic regulators in NAFLD. In the pathway and process enrichment analysis, chromatin organization and chromatin modifying enzymes were the most marked clusters, indicating the greater involvement of these proteins with HDMs in the regulation of gene expression.

Histone demethylation is a promising epigenetic target, and there are currently drugs being developed to target the HDMs [46]. A selective compound, GSK-J1, can inhibit KDM6B, thereby blocking the loss of H3K27me3 methylation with a half maximum inhibitory concentration (IC50) of 0.06 µM [47]. In addition, GSK-J1 also demonstrated inhibitory effects on KDM5C [48]. Kruidenier et al. have also developed a prodrug GSK-J4 with higher intracellular concentrations than GSK-J1 [47]. Another small molecule pan-JMJD inhibitor, JIB-04, has been demonstrated to exhibit inhibitory activity of demethylation of KDM6B in vitro, in cell lines, and in tumors in vivo as well as strong inhibitory activity against KDM4A (IC50 of 0.85 and 0.44 µM, respectively) [49]. Another compound, 2, 4-pyridinedicarboxylic acid (PDCA), was revealed as a potent inhibitor of KDM4A with an IC50 of 0.7 µM [50]. In light of the key role of histone demethylation in the epigenetic regulation of NAFLD, the development of these targeted agents would be attractive and promising.

Our study has several strengths. First, this is the first study to use bioinformatics exploration to reveal the profiles of histone demethylation in NAFLD, while we validated these results in in vitro experiments, albeit with disagreement. Second, we explored the expressions and prognostic values of DEGs in HCC in public databases and analyzed the pathways and network of interactions associated with these genes. However, our study was only validated in an in vitro model and lacked validation from in vivo experiments. Furthermore, our results show disparities with gene expression in human samples, but this could also indicate possible heterogeneity of gene expression at the research level. In the future, our team will employ transcriptomic analysis in in vivo experiments for further analysis.

## 5. Conclusions

We identified the histone demethylation profiles in NAFLD. Five HDMs, including KDM5C, KDM6B, KDM8, KDM4A, and JMJD7, were differentially expressed in NAFLD compared to healthy individuals, while no association of histone demethylation in fibrosis severity was revealed. However, in vitro studies presented inconsistent results, and future efforts are needed to confirm these findings in mouse models. KDM5C and KDM4A were significantly upregulated in HCC, while KDM8 expression was reduced, and they could be of prognostic values. Overall, these HDMs provide new insights into the pathogenesis of NAFLD and may be promising epigenetic targets.

## Figures and Tables

**Table 1:** Forward and reverse primer sequences of selected genes.

Genes	Forward Primer (5′-3′)	Reverse Primer (5′-3′)
KDM5C	ACTGCTGACCATTGCTGAACGC	CCTCCTTGAGAGCCTGGATGTT
KDM6B	GACCCTCGAAATCCCATCACAG	GTGCGAACTTCCACGGTGTGTT
KDM8	CACAGATGAGGAATGGTCCCAG	GCTGATGTCCTGCTTCAACTCC
KDM4A	TGCGGCAAGTTGAGGATGGTCT	GCTGCTTGTTCTTCCTCCTCATC
JMJD7	GGAGTCCTCTATGTGCAGAAGC	CAGCCAGAAGTTCACAGCATCG

## Data Availability

Publicly available datasets were analyzed in this study. This data can be found at the link in the text. Data from the in vitro studies in this study are available from the corresponding author upon request.

## Supplementary Materials

The following supporting information can be downloaded at: https://www.mdpi.com/article/10.3390/cimb45040237/s1. Figure S1. Volcano plots of DEGs from both databases. (A) SS vs. HC. (B) NASH vs. HC. (C) NASH vs. SS. (D) Advanced NAFLD vs. mild NAFLD. Abbreviations: DEGs, differentially expressed genes; SS, simple steatosis; HC, healthy control; NASH, nonalcoholic steatohepatitis; NAFLD, nonalcoholic fatty liver disease. Figure S2. Selected HDM gene expression in the GSE49541 database. Abbreviations: HDM, histone demethylase; NAFLD, nonalcoholic fatty liver disease; NS, not significant. An asterisk indicates that the *p*-value of < 0.05. Figure S3. Prognostic values of five HDM genes for disease-free survival in HCC. (A) KDM5C. (B) KDM6B. (C) KDM8. (D) KDM4A. (E) JMJD7. Abbreviations: HDM, histone demethylase; HCC, hepatocellular carcinoma; TPM, Transcripts Per Million; HR, hazard ratio. Figure S4. Networks and functional associations of selected HDM genes with the 20 strongest genes identified in the genemania database. Abbreviations: HDM, histone demethylase.
