# Supplementary material for: Histone Demethylation Profiles in Nonalcoholic Fatty Liver Disease and Prognostic Values in Hepatocellular Carcinoma: A Bioinformatic Analysis
Source: Curr Issues Mol Biol. 2023 Apr 20;45(4):3640–57. doi: 10.3390/cimb45040237 (PMC10136463; doi:10.3390/cimb45040237)
Supplement: Supplementary file 1 [file cimb-45-00237-s001.zip › cimb-2315112-supplementary.pdf]

Supplementary figures

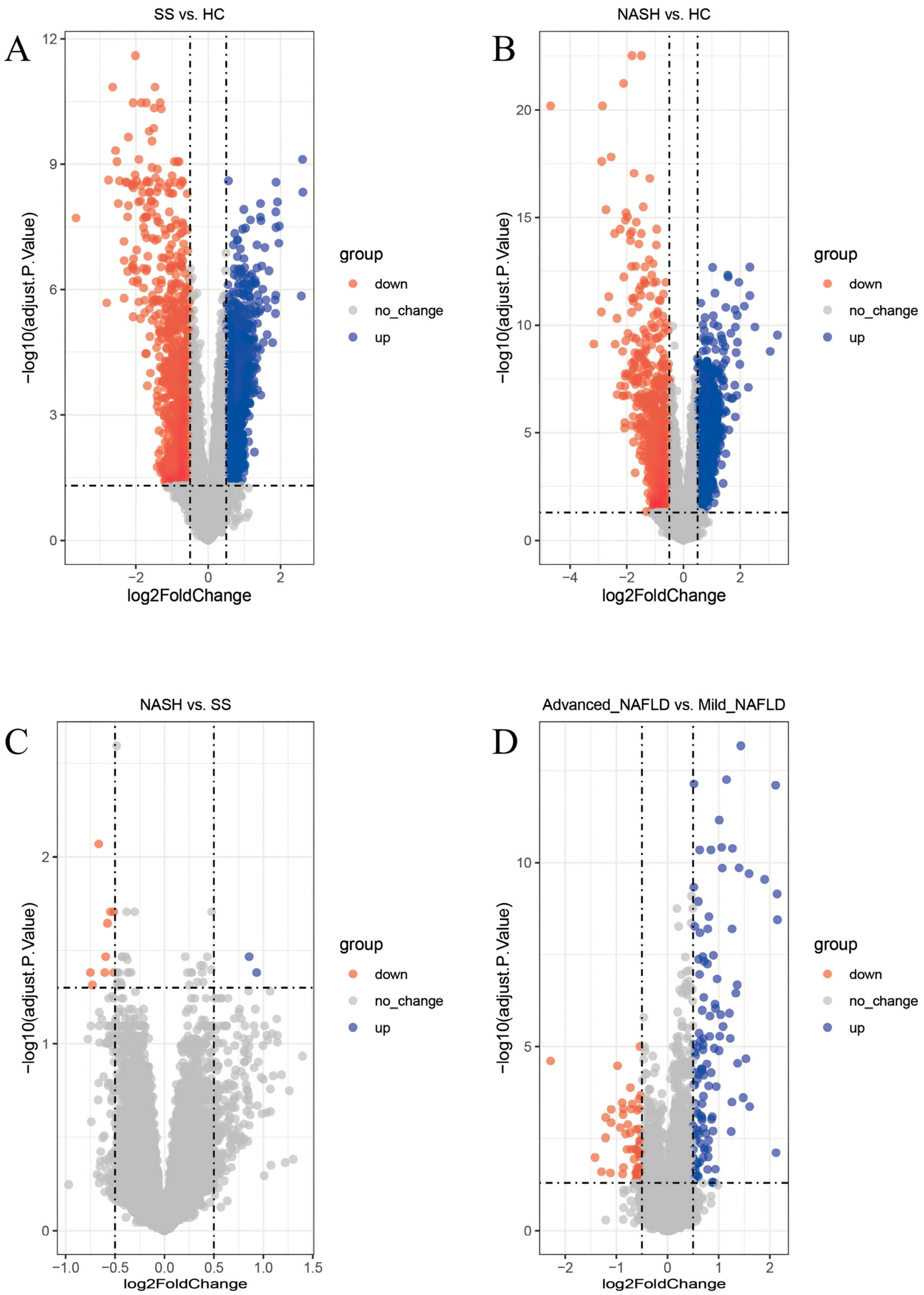

Figure S1. Volcano plots of DEGs from both databases. (A) SS vs. HC. (B) NASH vs. HC. (C) NASH vs. SS. (D) Advanced NAFLD vs. mild NAFLD. Abbreviations: DEGs, differentially expressed genes; SS, simple steatosis; HC, healthy control; NASH, non-alcoholic steatohepatitis; NAFLD, non-alcoholic fatty liver disease.

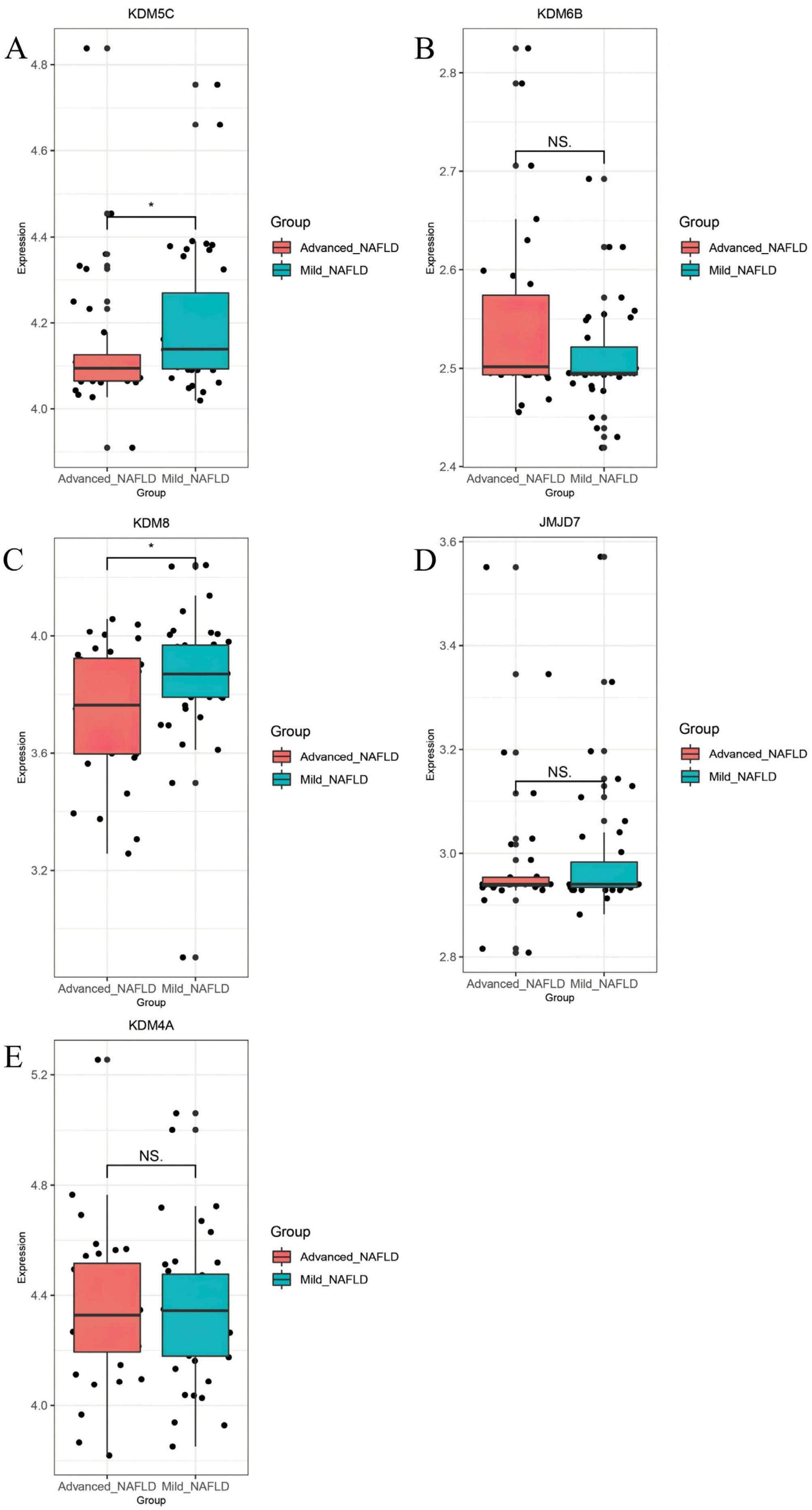

A
KDM5C
4.8
4.6
4.4
4.2
4.0
*
Expression
Advanced_NAFLD Mild_NAFLD
Group
Group
Advanced_NAFLD
Mild_NAFLD
B
KDM6B
2.8
2.7
2.6
2.5
2.4
NS.
Expression
Advanced_NAFLD Mild_NAFLD
Group
Group
Advanced_NAFLD
Mild_NAFLD
C
KDM8
4.0
3.6
3.2
*
Expression
Advanced_NAFLD Mild_NAFLD
Group
Group
Advanced_NAFLD
Mild_NAFLD
D
JMJD7
3.6
3.4
3.2
3.0
2.8
NS.
Expression
Advanced_NAFLD Mild_NAFLD
Group
Group
Advanced_NAFLD
Mild_NAFLD
E
KDM4A
5.2
4.8
4.4
4.0
NS.
Expression
Advanced_NAFLD Mild_NAFLD
Group
Group
Advanced_NAFLD
Mild_NAFLD

Figure S2. Selected HDM gene expression in the GSE49541 database. Abbreviations: HDM, histone demethylase; NAFLD, non-alcoholic fatty liver disease; NS, not significant. An asterisk indicates that the p-value of < 0.05.

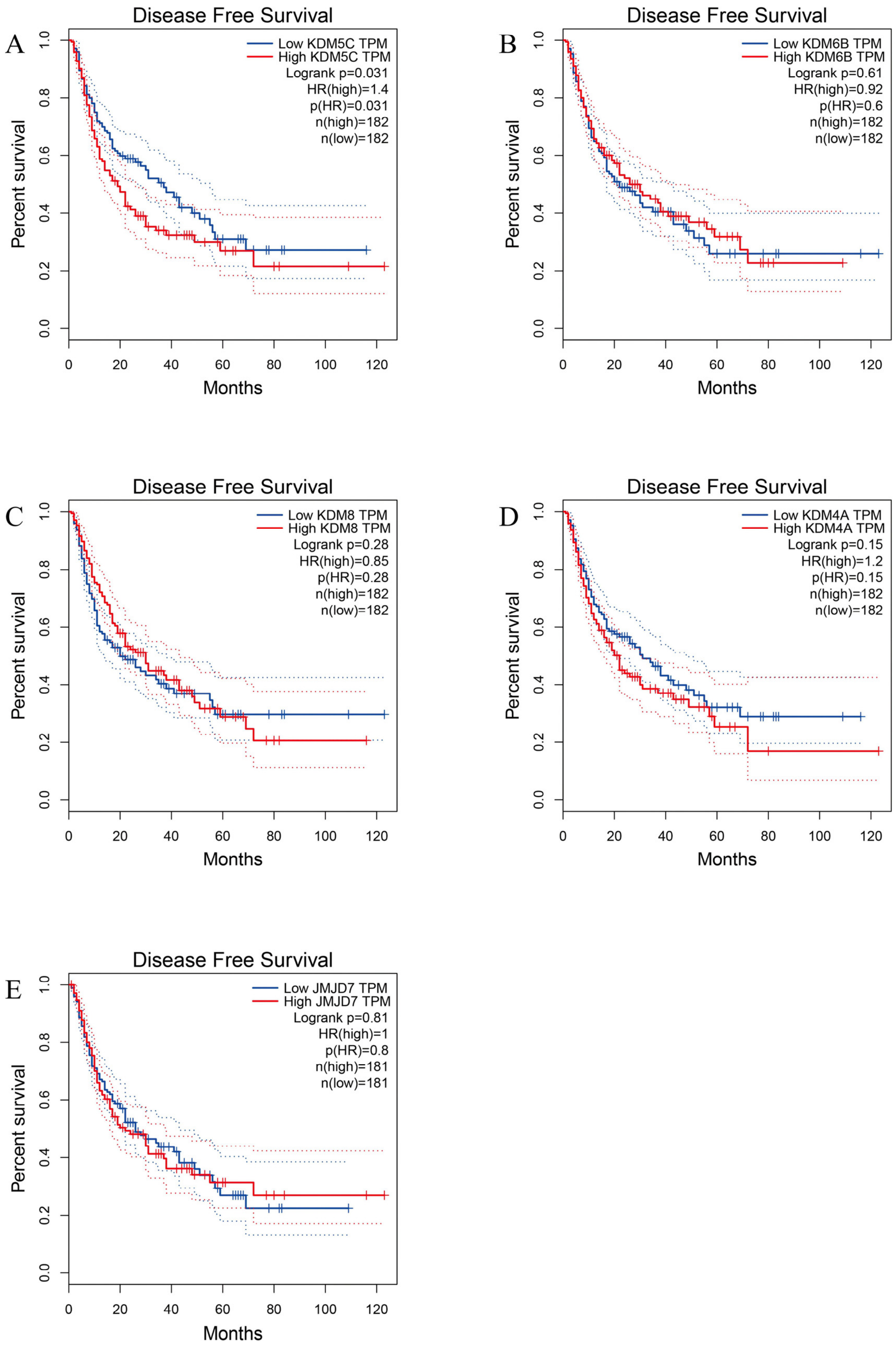
A
Disease Free Survival
Low KDM5C TPM
High KDM5C TPM
Logrank p=0.031
HR(high)=1.4
p(HR)=0.031
n(high)=182
n(low)=182
Percent survival
Months
B
Disease Free Survival
Low KDM6B TPM
High KDM6B TPM
Logrank p=0.61
HR(high)=0.92
p(HR)=0.6
n(high)=182
n(low)=182
Percent survival
Months
C
Disease Free Survival
Low KDM8 TPM
High KDM8 TPM
Logrank p=0.28
HR(high)=0.85
p(HR)=0.28
n(high)=182
n(low)=182
Percent survival
Months
D
Disease Free Survival
Low KDM4A TPM
High KDM4A TPM
Logrank p=0.15
HR(high)=1.2
p(HR)=0.15
n(high)=182
n(low)=182
Percent survival
Months
E
Disease Free Survival
Low JMJD7 TPM
High JMJD7 TPM
Logrank p=0.81
HR(high)=1
p(HR)=0.8
n(high)=181
n(low)=181
Percent survival
Months

Figure S3. Prognostic values of five HDM genes for disease-free survival in HCC. (A) KDM5C. (B) KDM6B. (C) KDM8. (D) KDM4A. (E) JMJD7. Abbreviations: HDM, histone demethylase; HCC, hepatocellular carcinoma; TPM, Transcripts Per Million; HR, hazard ratio.

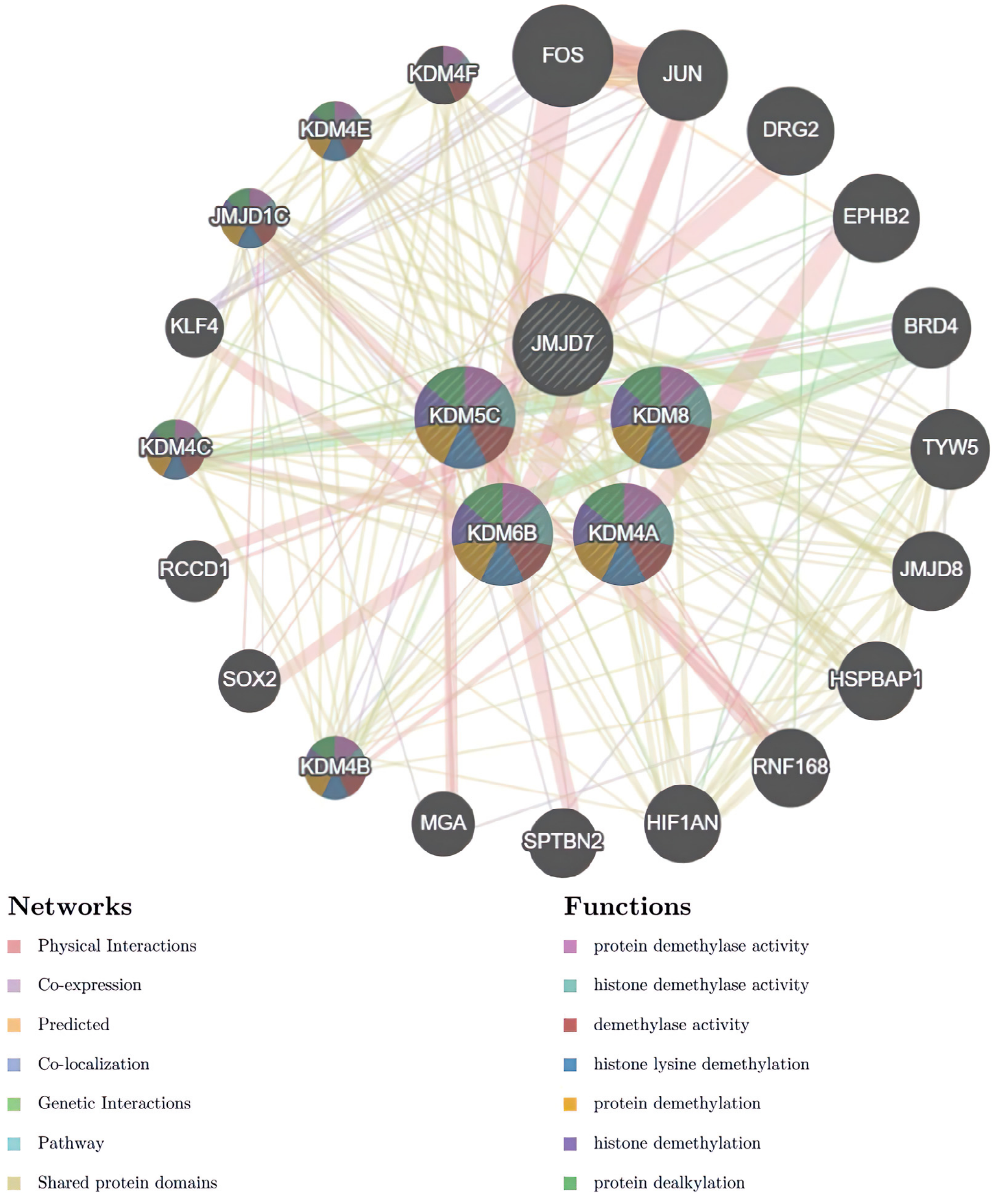

Figure S4. Networks and functional associations of selected HDM genes with the 20 strongest genes identified in the genemania database. Abbreviations: HDM, histone demethylase.
